# Eosinophilic Esophagitis in a Developing Country: Is It Different from Developed Countries?

**DOI:** 10.1155/2013/526037

**Published:** 2013-11-25

**Authors:** Abdulrahman Al-Hussaini, Toufic Semaan, Imad El Hag

**Affiliations:** ^1^University of King Saud bin Abdulaziz for Health Sciences, Children's Hospital, King Fahad Medical City, P.O. Box 59046, Riyadh, Saudi Arabia; ^2^Department of Medicine, King Saud Medical City, P.O. Box 7855, Riyadh, Saudi Arabia; ^3^Department of Pathology, Prince Sultan Medical City, P.O. Box 7855, Riyadh 111107, Saudi Arabia

## Abstract

*Background and Objective*. Despite the extensive reporting of eosinophilic esophagitis (EoE) from industrialized developed countries, reports from developing countries are rare. The aim of our study was to determine the epidemiological, clinical, and endoscopic features of EoE and response to therapy in children and adults from a developing country, Saudi Arabia. *Methods*. We identified patients diagnosed with EoE in our center from 2004 to 2011. EoE was defined as esophageal mucosal infiltration with a peak eosinophil count ≥15 eosinophils/high-powered field. *Results*. Forty-five patients were diagnosed with EoE (37 children and 8 adults; 36 males; median age 10.5 years, range from 1–37 years). Feeding difficulty, vomiting/regurgitation, and failure to thrive predominated in young children, whereas dysphagia and food impactions predominated in older children and adults. Allergy testing revealed food sensitization in 12 of 15 patients (80%); 3 responded to elemental formula, while 8 failed to respond to dietary manipulation after the allergy testing. Thirty-nine patients achieved remission by swallowed inhaled fluticasone. The majority of patients experienced a recurrence of symptoms upon the discontinuation of fluticasone. *Conclusion*. Our data indicate that EoE is increasingly recognized in Saudi Arabia and show many similarities to data from North America and Europe.

## 1. Introduction

In western countries, eosinophilic esophagitis (EoE) is one of the most common causes of intermittent solid-food dysphagia and food impaction in children and adults [1–3]. To our knowledge, in developing countries, data on EoE are scarce and have been limited to case reports and small case series [4–8]. Therefore, EoE epidemiology has yet to be well documented in developing countries and it remains to be determined whether the low number of reports from developing countries reflects true rarity of EoE or just under-recognition of the disease entity in this part of the world. We undertook this retrospective study in Riyadh, the capital city of Saudi Arabia, using this population of children and adults diagnosed with EoE, to clarify its epidemiology, clinical spectrum, relation to atopy, endoscopic and histological findings, and therapy. In this report, we will discuss similarities between EoE in Saudi Arabia and EoE reported from western countries. 

## 2. Methods and Patients

We conducted a retrospective study to identify all patients who were diagnosed in our institution with EoE from April 2004 to December 2011. Demographic data, clinical symptoms, history of atopic diseases, results of laboratory tests (including total blood eosinophil count, erythrocyte sedimentation rate, and albumin), endoscopic findings, histopathologic features, and results of treatment were collected and analyzed. Food impaction was defined as an event occurring after food ingestion during which solid food is retained in the esophagus and a visit to an emergency room for endoscopic removal was required. In young children, the term “feeding difficulty” was used to describe symptoms such as food refusal, oral aversion, gagging, and choking upon intake of solid food. Failure to thrive was defined as weighing less than the third percentile for each patient's age and sex. Peripheral eosinophilia was defined as >400 eosinophils/mm^3^. Data on serum immunoglobulin E levels, allergen-specific IgE test (RAST), and skin prick tests were collected when available. 

### 2.1. Diagnosis of EE

The diagnosis of EE was based on the demonstration of isolated eosinophilic infiltration of esophageal mucosa with ≥15 eosinophils per high power field (HPF) and remission of the disease on topical fluticasone or diets exclusion. Two biopsies each were obtained from the distal, upper, and mid-esophagus, as well as the antrum and the duodenum. All biopsies were fixed in formalin, embedded in paraffin, and stained with hematoxylin and eosin. The number of eosinophils in the most densely involved 400X microscopic HPF was counted, which included the eyepiece magnification, and the area of the microscopic field was equivalent to 0.22 mm^2^. Each esophageal specimen was evaluated for the presence of degranulated eosinophils (defined as free eosinophil granules in the esophageal epithelium), basal zone hyperplasia (BZH) (normal basal zone was defined as less than 25% of the total esophageal epithelium thickness in well oriented sections), and eosinophil clusters (defined as 5 or more eosinophils clustered together).

### 2.2. Allergy Testing

Hypersensitivity to common food and inhalant allergens was determined from patient history, serum immunoglobulin E levels, RAST, and skin prick tests. RAST was performed for milk, egg, soy, fish, peanut, and wheat using UniCAP platform. IgE antibody was classified as a negative or positive test result (CAP > 0.35 kUA/L). Patients underwent skin prick test to foods based on their clinical history, in addition to a standard panel of foods and inhalants. Skin prick testing was performed by puncturing the forearm with a bifurcated needle (Allergy Labs of Ohio, Columbus) and introducing a purified commercial allergen preparation (Greer Laboratories, Lenoir, NC) to be absorbed through the skin. Reactions were recorded by measuring the diameter in millimeters of the largest wheal and flare at 15 minutes. Testing was considered positive if the wheal was 3 mm greater than the negative control. The diagnoses of asthma, allergic rhinitis, and atopic dermatitis were based on physician diagnosis.

### 2.3. Treatment

The choice of therapy was left to the discretion of the treating gastroenterologist.

*Medical Therapy. *Patients were treated with swallowed aerosolized fluticasone propionate from a metered dose inhaler at a dose of 250 micrograms twice daily for children <10 years of age and 500 mcg twice daily for children >10 years of age and adults. This dose was dispensed for 2 months before starting to taper the dose to 0 over the next 2 months. In practice, patients were instructed to swallow the agent, which was sprayed into the mouth with a metered dosed inhaler without a spacer, and not to eat or drink for at least 30 min after administration. Patients were advised to rinse their mouths out with water to prevent oral candidiasis.

*Dietary Treatment*. Dietary therapy was defined as either “allergy testing guided-dietary restriction,” in which selected foods were excluded based on the results of allergy testing, or “complete dietary elimination,” in which an amino acid-based milk formula was started. If no foods were identified by allergy testing or no response was observed from dietary therapy, a medical therapy of swallowed fluticasone inhaler was begun. A repeat upper endoscopy was offered to all patients at 6–8 weeks after therapy was initiated. 

### 2.4. Definition of Remission, Relapse, and Treatment Failure

Patients with mean eosinophil count 0 to 5/HPF in the repeated esophageal biopsies after 6–8 weeks of therapy were considered to be in remission. Those patients with mean eosinophil count 5–14/HPF and improvement of symptoms were considered to be in partial remission. An eosinophil count ≥15/HPF was defined as treatment failure. 

The study was approved by the Local Review Board in King Fahad Medical City (IRB Log number 12-218) and had been performed in accordance with the ethical standards laid down in the 1964 Declaration of Helsinki and its later amendments.

### 2.5. Statistical Analysis

The data were analyzed using SPSS Pc+ version 16.0 (Chicago, USA) statistical software. Descriptive statistics (mean, standard deviation, and percentages) were used to describe the quantitative and categorical variables. Student's *t*-test for independent samples was used to compare the mean values of the quantitative variables. Pearson's Chi-squared test was used to test any association between the two categorical variables. Fisher's exact test was used for small samples. A *P* value < 0.05 was considered statistically significant.

## 3. Results

### 3.1. Epidemiologic Characteristics

**Figure 1:**
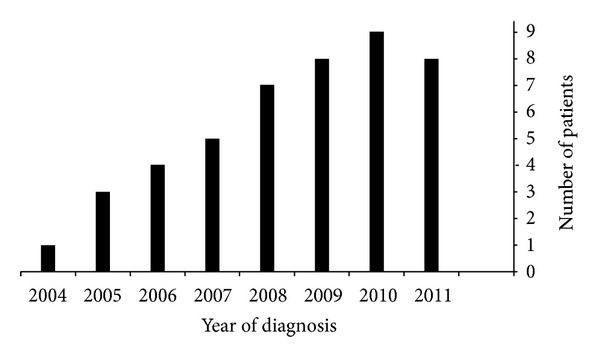
Distribution of eosinophilic esophagitis cases by year of diagnosis.

From April 2004 to December 2011, 45 patients were diagnosed with EoE (37 children and 8 adults). The mean age at the diagnosis of EoE, the mean duration of symptoms prior to the diagnosis, and the male to female ratio are shown in Table 1.  Figure 1 demonstrates an increasing number of new cases of EoE over the study period. A family history of atopy and at least one concomitant allergic disease were found in the majority of the patients (39/45; 86%) (Table 1).

### 3.2. Clinical Characteristics of EoE Patients

Table 1 shows the presenting symptoms of EoE. Patients were categorized into two separate subgroups: the pediatric group (<18 years) and the adult group (≥18 years). Dysphagia to solid food was the most common presenting symptom in both children (80%) and adults (100%). Clinical presentation of EoE in adults was limited to three main symptoms: dysphagia, food impaction, and heartburn, while the spectrum of presenting symptoms in children was more varied and included dysphagia, vomiting, failure to thrive, food impaction, heartburn, and abdominal pain. Among these presenting symptoms of EoE, only heartburn was statistically more frequent in adults (62.5% versus 11%, *P* = 0.005). An analysis of the frequency of presenting symptoms based on age at presentation (Table 2) shows the predominance of vomiting and failure to thrive in young children (age ≤5 years, mean 3.3 ± 1.5) compared to older children and adolescents. On the other hand, dysphagia and food impaction predominated in older children and adolescents. Eight of 12 young children and 4 of the adults had received antigastroesophageal reflux disease (GERD) therapy prior to diagnosis of EoE. The anti-GERD therapy included omeprazole and domperidone. Three children were diagnosed with EoE incidentally: 3- and 9-year-old boys underwent upper endoscopy for removal of a hair clip and a glass piece stuck in the esophagus, respectively, and an 11-year-old boy with known type 1 diabetes had endoscopy for screening after a positive celiac profile. Findings from the esophageal endoscopies suggestive of EoE led to multiple biopsies at different levels of the esophagus. Although the three children and their parents initially denied any upper gastrointestinal symptoms, upon further direct questioning after-endoscopy, they reported mild intermittent difficulty on solid food intake or eating behaviors (requiring water when eating or slow feeding) that gave indirect clues about dysphagia.

### 3.3. Endoscopic and Histopathological Findings

Loss of vascular pattern and furrowing of esophageal mucosa were the two most frequent endoscopic findings of EoE in our pediatric and adult patients (95%) followed by ring formation (35%), whitish exudates (21.5%), mucosal ulcer and polypoid lesions (9% each), and upper esophageal strictures (4.5%). The esophagus looked macroscopically normal in 2 children (4.5%). Analysis of the frequency of the endoscopic and histopathologic features of EoE showed similar rates among children and adults with the exception that esophageal ring formation occurs significantly more in adults compared to children (75% versus 27%, *P* = 0.03) (Table 3). Gastric and duodenal biopsies were negative for eosinophilic infiltration.

### 3.4. Allergy Study

Fifteen children (33%) underwent allergy testing using total IgE level, RAST, and a skin prick test (Table 4). Three children were negative for all three tests (20%). Total serum IgE was elevated in 9 patients (60%) (mean: 387 Ku/mL; range: 278–1133 Ku/mL; normal <100 Ku/mL). Specific IgE for the suspicious food (RAST) was positive in 12 patients (80%): 4 with a positive test for a single food, 2 for 2 food allergens, and 6 for ≥3 food allergens. The most common food allergen was milk (8 patients), followed by egg (6 patients) and soybean and wheat (5 patients each). The skin prick test was positive in 7 of 11 patients on whom the test was performed: 4 showed positivity for food allergens only, 2 for foods and aeroallergens, and 1 for aeroallergens only. Ten out of 12 patients who underwent both RAST and the skin prick test received the same positive or negative result on both (83%).

### 3.5. Treatment and Outcome

**Figure 2:**
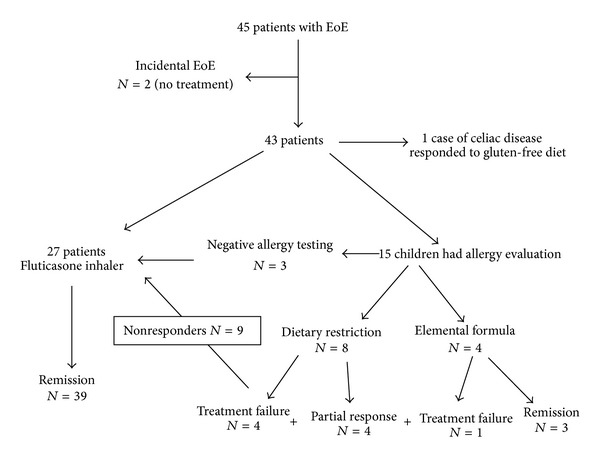
Diagram of 45 patients with eosinophilic esophagitis: treatment and outcome.

Figure 2 summarizes the treatment and outcome of the 45 patients. Following the previously described protocol, the 12 patients who tested positive for allergies were initially treated with dietary therapy. Because of the poor palatability of elemental formulas, elimination diets based on the allergy testing were prescribed for 8 older children; the remaining 4 younger children (≤2 years) were prescribed an exclusive elemental formula. Three of the 4 young children responded to the exclusive elemental milk formula and 1 did not respond due to noncompliance. Four of the eight older children on the elimination diet achieved partial remission and the other 4 did not respond. Three children with negative allergy tests for food sensitization were recommended a swallowed fluticasone inhaler at the time of their diagnosis of EoE. In total, 39 patients with EoE (86%) received the swallowed fluticasone inhaler and all went into remission. The two children with proximal esophageal strictures underwent endoscopic dilation to relieve their dysphagia. The parents of the 2 children with incidentally diagnosed EoE opted not to receive any therapy. The child with celiac disease achieved remission of EoE, as evidenced by repeated esophageal biopsies 6 months after initiation of a gluten-free diet. Only 2 of the 39 patients developed oral thrush, which was treated by oral nystatin. Two children underwent dilation of esophageal strictures using Savary dilators.

Four of the 45 patients were lost to follow-up. Adherence to the amino acid-based formula was not sustainable in 3 young children, whose symptoms relapsed upon the reintroduction of solid food. The remaining 38 patients with available follow-up data reported relapse of their symptoms within 3 to 9 months after stopping therapy, which required them to restart fluticasone therapy. After achieving remission, those patients required a maintenance dose ranging from 250 mcg/day (in children ≤10 years of age) to 500 mcg (in children >10 years of age and adults) to maintain remission. The follow-up period ranged between 0.5 to 7 years (median 3.2 years; mean 3.1 ± 1.6 years). No adverse effects on patient growth have been observed.

## 4. Discussion

Our data indicate that EoE is increasingly recognized in Saudi Arabia. However, it is not clear if this dramatic increase in number of EoE cases diagnosed per year is due to an escalating epidemiology of EoE in Saudi Arabia or to increased awareness of this entity, or both. It is possible that the rise in EoE in the Saudi community is related to the concurrent increase in the prevalence of other atopic diseases such as asthma and atopic dermatitis [9]. Our data on EoE show many similarities to data from North America and Europe: male predominance (male : female ratio = 4 : 1), a majority of patients who are atopic (75%), characteristic endoscopic and histopathologic findings, the chronicity and relapsing nature of EoE, and the effectiveness of a swallowed fluticasone inhaler in treating EoE.

The results of our study show that EoE predominantly followed two distinct patterns of clinical presentation that varied with age. Young children with EoE presented with feeding difficulty, vomiting/regurgitation, and failure to thrive, whereas older children, adolescents, and adults were more likely to present with dysphagia and food impactions. Due to the similar clinical presentations of GERD and EoE in young children, the distinction between these 2 entities can be difficult. Many of the young children in our study had received conventional anti-GERD therapy, including proton pump inhibitors (PPI), prior to the diagnosis of EoE. In fact, some patients with EoE underwent fundoplication to treat severe GERD [10]. This emphasizes the need for an esophageal biopsy when controlling GERD-like symptoms becomes difficult. 

EoE in older children and adults typically presents with dysphagia and esophageal food impaction in an episodic manner [11]. The findings from our study, in combination with a review of the medical literature, suggest that EoE typically affects young adult men who have allergic predisposition [11]. Heartburn was a more common symptom in our adult patients at 62%, compared to 11% in children (*P* = 0.005). This difference in the frequency of heartburn may be related to the younger patients' limited expressive language ability to communicate discomfort and to localize pain or discomfort. The duration of symptoms prior to the diagnosis of EoE was significantly longer in adults compared to children (3.2 years versus 1.1 years). This finding could be attributed to milder nature of EoE in adults or to the ability of adults to rapidly adapt their eating habits to manage their swallowing difficulties. A number of these compensatory behaviors will escape detection unless the clinician maintains a high index of suspicion or the adult patient presents to the emergency department with food impaction.

Asymptomatic subjects who were incidentally discovered in our study would have remained undiagnosed had endoscopic examination with esophageal biopsies not been performed. Many of the initial symptoms of EoE are modest and known only to the child. In children with atopy being treated in allergy clinics, the symptoms of EoE may be overshadowed by symptoms of primary atopic disease. Therefore, the diagnosis of EoE may be overlooked for several years before their gastrointestinal symptoms grow severe enough that parents seek medical advice and require referral to a pediatric gastroenterologist or that the child presents to the emergency department with food impaction. Thus, we think that EoE may be an undiagnosed or unrecognized condition, but not an uncommon one.

The natural history of EoE is not well defined yet; however, the predominance of the two patterns of symptoms in 2 different populations (young children versus older children and adults), that have been observed in previous studies [12–15], suggests potential progression of an untreated disease. One possible analysis of these findings is that the esophagus in EoE passes through 2 phases, an “inflammatory phase” in infants and young children and a “fibrotic phase” in older children. The first phase is characterized by extensive eosinophilic inflammation that causes damage to the esophagus and its sphincter, resulting in reflux symptoms. As the disease progresses in untreated cases, the esophagus proceeds into a “fibrotic phase,” characterized by sub-epithelial fibrosis and subsequent esophageal remodeling and thickening [16, 17]. This thickening of the esophagus leads to narrow esophagus, swallowing difficulty, and eventually food impaction. The presence of 2 children (4.5 and 10 years old) with esophageal strictures in our study group suggests that this progressive process may evolve rapidly in some children. An alternative explanation is that there are different phenotypes of EoE. Natural history studies are needed to define the long-term risks of EoE as the disease progresses and the consequences of this esophageal inflammation.

As much as 95.5% of the EoE patients in our study cohort displayed one of the endoscopic features that have been identified as characteristic for EoE [18]. In the appropriate clinical setting, the identification of any of the endoscopic features described in Table 3 supports but does not confirm the diagnosis of EoE [19]. Occasionally, the endoscopic appearance of the esophageal mucosa in EoE may be normal or characterized by only minimal changes [20]. We had 2 children (4.5%) with esophageal mucosa that appeared normal on endoscopy. Although it is possible that subtle esophageal findings may not have been identified by the endoscopist, the lack of endoscopic findings in the presence of clinical symptoms and of histopathologic conditions typical of EoE reinforces the need for biopsies from the proximal and distal esophagus in patients with dysphagia or GERD-like symptoms, as endorsed in the recent consensus guidelines for EoE [19].

Fluticasone propionate, when swallowed from a metered-dose inhaler, improves the clinicopathologic features of EoE in most patients [21]; however, when discontinued, the disease almost always recurs [13, 22]. The chronic and relapsing nature of EoE observed in our patients has been described before in pediatric and adult patients for 8 and 11 years of follow-up, respectively [22, 23]. When topical steroids are used chronically, growth and the development of side effects should be carefully monitored in children. The lack of long-term safety data remains a relative concern. Further studies are needed to clarify the most effective topical steroid dose required for the initial disease treatment and for maintenance therapy in both children and adults. Before 2012, it was not our practice to initially treat isolated esophageal eosinophilia with PPI for 8–12 weeks, as endorsed in the recent consensus guidelines for EoE [19] to exclude a recently described subgroup known as PPI-responsive esophageal eosinophilia or PPI-responsive EoE. Therefore, we have not been able to identify PPI-responsive esophageal eosinophilia/PPI-responsive EoE patients in our cohort, an important limitation in our study. However, the response of our patients to topical fluticasone or dietary exclusion, one of the diagnostic criteria endorsed in the recent consensus guidelines for EoE, confirms the diagnosis of EoE in our patients. 

In conclusion, we have seen a steady increase in the number of new cases of EoE on an annual basis in the Saudi population. The results of this study suggest that EoE should be considered in the differential diagnosis of an atopic child presenting with GERD-like symptoms or dysphagia. In this retrospective study with a follow-up period up to 7 years, we determined that EoE is a chronic relapsing condition.

## Figures and Tables

**Table 1:** Clinical characteristics of patients with eosinophilic esophagitis.

Variable	Pediatric group (*N* = 37)	Adult group (*N* = 8)	*P* value
Mean age at presentation (year)	8 ± 4.2 (range 1–17 )	22.5 ± 18.3 (range 18–37 )	
Sex (M/F)	30/7 (4.2 : 1)	6/2 (3 : 1)	0.92
Mean duration of symptoms before diagnosis (year)	1.16 ± 1	3.2 ± 1.5	0.0001
Presenting complaints			
Vomiting	9 (24%)	0	0.28
Failure to thrive	9 (25%)	0	0.28
Dysphagia/feeding difficulty	31 (84%)	8 (100%)	0.52
Food impaction	11 (30%)	5 (62.5%)	0.18
Heart-burn	4 (11%)	5 (62.5%)	0.005
Abdominal pain	4 (11%)	0	0.77
Incidental finding of EoE	3 (8%)	0	0.96
Personal history of atopy			
Asthma	18 (48.5%)	2 (25%)	0.41
Eczema	8 (21.5%)	1 (12.5%)	0.92
Rhinitis	4 (11%)	6 (75%)	0.0005
Family history of atopy	31 (84%)	8 (100%)	0.52
Peripheral eosinophilia	20 (54%)	2 (25%)	0.27

**Table 2:** Symptoms of eosinophilic esophagitis based on age at presentation.

Symptom	≤5 years (*N* = 12)	6–10 years (*N* = 13)	11–15 years (*N* = 10)	>15 years (*N* = 10)
Dysphagia/feeding difficulty	7 (41%)	12 (92%)	10 (100%)	10 (100%)
Vomiting	8 (67%)	1 (7.5%)	0	0
Failure to thrive/weight loss	7 (41%)	2 (15.5%)	0	0
Heart-burn	0	1 (7.5%)	3 (30%)	5 (50%)
Esophageal food impaction	2 (17%)	4 (31%)	4 (40%)	6 (60%)
Abdominal pain	0	2 (15.5%)	2 (20%)	0

**Table 3:** Endoscopic and histopathological features of 45 patients with eosinophilic esophagitis.

	Pediatric group (*N* = 37)	Adult group (*N* = 8)	*P* value
Esophageal endoscopic findings			
Loss of vascular markings	35 (94.5%)	8 (100%)	0.78
Furrowing	34 (92%)	8 (100%)	0.96
Whitish exudates	8 (21.5%)	0	0.35
Ring formation	10 (27%)	6 (75%)	0.03
Ulcer	4 (11%)	0	0.77
Stricture	2 (5.4%)	0	0.78
Polypoid lesion	2 (5.4%)	2 (25%)	0.28
Normal esophagus	2 (5.4%)	0	0.78
Esophageal histopathological findings			
Eosinophilic microabscess	9 (24.4%)	0	0.28
Eosinophilic degranulation	30 (81%)	8 (100%)	0.42
Basal cell hyperplasia	35 (94.5%)	7 (87.5%)	0.78
Papillae elongation	30 (81%)	6 (75%)	0.66

**Table 4:** Results of allergy testing and therapy in 15 children with eosinophilic esophagitis.

Patient	Ag (yr)	IgE (<100 ku/mL)	Positive RAST	Positive SPT	Action	Outcome
1	5	604	Milk, egg, soy	Milk, wheat, egg, mites, pollens, molds	Dietary restriction	Treatment failure
2	11	46	Tuna, tomato	Negative	Dietary restriction	Partial response
3	1.5	309	Milk, egg, soy, wheat, nut	Milk, egg, soy, wheat, nut	Elemental formula	Remission
4	5	267	Milk	Negative	Dietary restriction	Partial response
5	1	983	Egg and milk	Milk, egg, soy	Elemental formula	Remission
6	11	2	Negative	Mites, pollens	Fluticasone	Remission
7	2	57	Milk	Milk	Elemental formula	Remission
8	10	602	Wheat, nuts and soybean	Pollens, weeds, mites, nut, sesame, almond	Dietary restriction	Partial response
9	8	1133	Egg, soybean, wheat, milk	ND	Dietary restriction	Treatment failure
10	1.5	37	Milk	ND	Elemental formula*	Treatment failure
11	5	1008	Milk, wheat, and peanut	ND	Dietary restriction	Treatment failure
12	4	19	Negative	Negative	Fluticasone	Remission
13	5	39	Negative	Negative	Fluticasone	Remission
14	4	278	Egg	Egg	Dietary restriction	Partial response
15	4	433	Egg, wheat, soybeans, nuts	ND	Dietary restriction	Treatment failure

N: normal; ND: not done; RAST: radioimmunoassay test; SPT: skin prick test; yr: year; *child was not compliant on elemental formula.
